# Evaluation of Birth Weight and Neurodevelopmental Conditions Among Monozygotic and Dizygotic Twins

**DOI:** 10.1001/jamanetworkopen.2023.21165

**Published:** 2023-06-30

**Authors:** Johan Isaksson, Vladislav Ruchkin, Therese Ljungström, Sven Bölte

**Affiliations:** 1 Child and Adolescent Psychiatry Unit, Department of Medical Sciences, Uppsala University, Uppsala, Sweden; 2 Center of Neurodevelopmental Disorders, Centre for Psychiatry Research, Department of Women’s and Children’s Health, Karolinska Institutet and Stockholm Health Care Services, Stockholm, Sweden; 3 Child Study Center, Yale University School of Medicine, New Haven, Connecticut; 4 Sala Forensic Psychiatric Clinic, Sala, Sweden; 5 Curtin Autism Research Group, School of Occupational Therapy, Social Work and Speech Pathology, Curtin University, Perth, Australia; 6 Child and Adolescent Psychiatry, Stockholm Health Care Services, Stockholm, Sweden

## Abstract

**Importance:**

Low birth weight is associated with an increased likelihood of neurodivergence and neurodevelopmental conditions (NDCs) such as autism, attention-deficit/hyperactivity disorder (ADHD), and intellectual disability. However, it is unclear whether birth weight contributes independently to NDCs or whether the association is predominantly driven by genetic predisposition.

**Objective:**

To estimate the associations between birth weight and dimensional (trait) and categorical (diagnoses) NDC outcomes, while adjusting for genetic risks.

**Design, Setting, and Participants:**

A co-twin design was applied to this case-control study conducted in Sweden. Diagnostic assessments were conducted between August 2011 and March 2022, within the Roots of Autism and ADHD Twin Study in Sweden (RATSS) during a 2.5-day participant visit to the clinic. The RATSS sample comprised phenotyped monozygotic and dizygotic twins enriched for NDCs. Data analysis was conducted in November 2022.

**Exposure:**

Birth weight.

**Main Outcomes and Measures:**

Categorical and dimensional operationalizations of autism, ADHD, and intellectual disability were assessed. Generalized estimating equation models were fitted across and within twin pairs.

**Results:**

The study sample included 393 twins: 230 were monozygotic and 159 were dizygotic (zygosity was unknown for 4). Their median age was 15 (range, 8-37) years. There were 185 female participants (47.1%) and 208 male participants (52.9%). Across twin pairs, higher birth weight was associated with fewer autistic traits (unstandardized β [B], −5.51 [95% CI, −10.09 to −0.94]) and lower odds of autism diagnosis (OR, 0.63 [95% CI, 0.45 to 0.88]) and intellectual disability (OR, 0.42 [95% CI, 0.19 to 0.92]). Within pairs, the association between birth weight and dimensional autism (B, −17.35 [95% CI, −28.66 to −6.04]) and categorical autism (OR, 0.02 [95% CI, 0.001 to 0.42]) remained among monozygotic pairs but not dizygotic pairs. In addition, higher birth weight was associated with lower odds of ADHD diagnosis (OR, 0.003 [95% CI, 0 to 0.70]), fewer ADHD traits (B, −0.25 [95% CI, −0.39 to −0.11]), and higher IQ ratings (B, 7.43 [95% CI, 1.05 to 13.82]) among monozygotic twins.

**Conclusions and Relevance:**

The findings of this co-twin study suggest an association between low birth weight and NDCs, but they also acknowledge the importance of genetics because the associations observed were only statically significant among monozygotic twins. It is of pivotal importance to facilitate early identification of factors contributing to fetal growth restriction to minimize detrimental outcomes.

## Key Points

**Question:**

After adjustment for genetic factors, is birth weight associated with neurodevelopmental conditions?

**Findings:**

In this case-control study of 393 twins in Sweden, the twin with a lower birth weight in monozygotic twin pairs, but not dizygotic pairs, had more autism and attention-deficit/hyperactivity disorder (ADHD) symptoms, lower IQ ratings, and higher odds of having a diagnosis of autism and ADHD compared with their co-twin.

**Meaning:**

These findings suggest that birth weight contributes to neurodevelopmental conditions when adjusting for genetic factors.

## Teaser

This case-control study estimates the associations between birth weight and neurodevelopmental conditions, such as autism, attention-deficit/hyperactivity disorder, and intellectual disability, among monozygotic and dizygotic twins.

## Introduction

Neurodevelopmental conditions (NDCs) are present in approximately 18% of the general US population.^1^ They include autism, attention-deficit/hyperactivity disorder (ADHD), and intellectual disability (ID), among other diagnoses. Neurodevelopmental conditions are heterogenous in causation, neurobiology, and behavioral phenotypes; they tend to co-occur and increase susceptibility for mental and somatic health issues.^2,3^

Although NDCs have a strong genetic component, nonshared environmental (NSE) factors also play a substantial role in their etiology.^3,4^ Nonshared environmental factors are exposures that are unique to members within the same family and make them differ from each other. Low birth weight is a specific NSE factor that has been linked to autism,^5^ ADHD,^6^ and low IQ^7^ and may be associated with long-lasting outcomes in child development and health.^8^ However, the origins of the association between low birth weight and NDCs remain difficult to establish, owing to potential confounding factors. It is unclear whether low birth weight independently contributes to NDCs or if its association with NDCs can be better explained by factors such as genetic susceptibility to both low birth weight and NDCs. By applying a twin co-control design using dizygotic and monozygotic twins, it is possible to approach this research question because dizygotic twins share approximately 50% of their genome (and all shared environment), whereas monozygotic twins share 100% of their genome. If the association between birth weight and NDCs remains among twin pairs, then NSE factors specific to each fetus should be directly involved in the underlying pathway of NDCs.

Although previous research provides some support for a direct contribution of low birth weight to NDCs,^4^ the number of twin studies exploring the issue has been limited. Willfors et al^9^ reported a trend in which lower birth weight was associated with autism and autistic traits within twin pairs. Losh et al^10^ found that among twin pairs discordant for autism, the lower-weighted twin had higher odds of being categorized as having autism; however, a limitation was that autism diagnosis was based solely on a parent questionnaire and not on a full-scale diagnostic assessment. Interestingly, the odds of being categorized as autistic for the lower-weighted twin were similar for monozygotic and dizygotic twin pairs,^10^ which suggests a direct contribution of NSE factors to autism and not a genetic contribution to the association. For ADHD, Lehn et al^11^ conducted a twin study operationalizing the condition based on diagnostic interviews and ratings. They found that within monozygotic twin pairs, individuals with ADHD had lower birth weight compared with their co-twin without ADHD. This finding suggests a direct contribution of NSE factors. Studies using dimensional operationalizations of ADHD have produced mixed findings, with both reported associations^12,13,14,15^ and no associations^16^ in population-based cohorts. Reported within-pair differences remained in some studies for both monozygotic and dizygotic subsets, suggesting a direct contribution of NSE factors^12,14^; in another study, they only presented among monozygotic twins, although with similar estimates.^13^ Additionally, previous studies have reported no associations between lower birth weight and ID,^17^ while associations have been reported for lower IQ.^18,19^

A recent study found that in pairs with birth weight discordance of 18% or greater, the lower-weighted twin had a higher likelihood of presenting with traits of autism and ADHD than their co-twin.^20^ However, that study only included monozygotic twins and was limited to few discordant pairs and a categorical measure of birth weight discordance. There are also few studies exploring differences in the association between birth weight and dimensional and categorical NDCs stratified by zygosity. Birth weight discordance within monozygotic twins is more likely to reflect differences in placental function or blood and nutrient supply to the individual fetus than shared genetic or environmental factors. Unequal placental sharing is regarded as a major cause of fetal growth discordance in monozygotic twins, and, in extreme cases, an unbalanced intertwin blood transfusion may lead to twin-to-twin transfusion syndrome (TTTS), in which 1 twin gives away more blood than they receive, with increased risk of malnourishment and a wide range of detrimental outcomes.^21^

With the objective to expand knowledge within the field, we applied a co-twin control design within a sample of deeply phenotyped monozygotic and dizygotic twins enriched for NDCs. To enable estimation of the associations between birth weight discordance and neurodivergence for both clinical and typical phenotypes, dimensional (trait) and categorical (diagnoses) outcomes were assessed. We hypothesized that (1) birth weight difference is associated with clinical and trait-level NDCs and (2) the association between birth weight differences and NDCs would remain within twin pairs even after adjusting for familial confounders, indicating a direct association between low birth weight and neurodivergent phenotypes.

## Methods

### Procedure and Participants

Twins were assessed between August 2011 and March 2022 within the Roots of Autism and ADHD Twin Study in Sweden (RATSS). All Swedish twin pairs are eligible to participate in the RATSS if 1 twin or both twins obtain a positive screening result for NDCs or typical development.^20,22^ A subsample of monozygotic twins was included in a previous publication.^20^ This study adds to the previous research (1) by including a larger sample of twins and (2) by comparing the link between low birth weight and NDCs in both monozygotic and dizygotic twins. Zygosity was determined with a panel of 48 single-nucleotide polymorphisms.^23^ In a few cases in which DNA results had not yet been analyzed (n = 15 pairs), a 4-item zygosity questionnaire based on physical appearance was used instead. We lacked data on zygosity for 4 participants. This study was approved by the National Swedish Ethical Review Board. All participants and/or their legal guardians provided written and oral informed consent. The study conformed to the Strengthening the Reporting of Observational Studies in Epidemiology (STROBE) reporting guideline.

### Measures

A group of clinicians performed the diagnostic assessment according to *Diagnostic and Statistical Manual of Mental Disorders* (Fifth Edition) criteria during a 2.5-day participant visit to the clinic, using diagnostic interviews, medical history records, and standardized diagnostic tools. Diagnostic tools included the Autism Diagnostic Observation Schedule–2, the Autism Diagnostic Interview–Revised, the Diagnostic Interview for ADHD in Adults, the Structured Clinical Interview for *DSM-IV*, and the Adaptive Behavior Assessment System–2. (Additional information can be found in Bölte et al.^22^)

Autistic traits were assessed with parent ratings on the Social Responsiveness Scale–2 (SRS-2). The SRS-2 targets interpersonal behavior, communication, and restricted, inflexible, and repetitive interests and behaviors. The scale includes 65 items and is rated on a 4-point Likert-type scale (0, not true; 1, sometimes true; 2, often true; and 3, almost always true), with a total score ranging between 0 and 195. A higher SRS-2 score indicates more autistic traits.

Attention-deficit/hyperactivity disorder traits were assessed with the parent-rated attention subscale from the Child Behavior Checklist (CBCL) and the Adult Behavior Checklist (ABCL), depending on participant age. Items in the CBCL and ABCL are rated on a 3-point Likert-type scale (0, not true; 1, sometimes true; and 2, very often true). The attention subscale includes 10 items in the CBCL and 17 items in the ABCL. In this study, CBCL and ABCL attention total scores were calculated as mean values, with a higher mean indicating more ADHD symptoms.

Intelligence quotient ratings were assessed with the General Ability Index (GAI) of the Wechsler Intelligence Scale for Children–IV or the Wechsler Adult Intelligence Scale for Adults–IV, depending on participant age. The GAI is a full score measure that provides an estimate of intellectual functioning less influenced by processing speed and working memory, which are often compromised by NDCs. The GAI provides a composite score based on 3 verbal comprehension subtests and 3 perceptual reasoning subtests.

### Birth Weight

The RATSS parent-report questionnaire was designed to cover prenatal, perinatal, and postnatal factors and include information about a twin’s birth weight. The questionnaire has been validated against medical registry data and was completed during the participant visit to the clinical research unit.^9^ There was high agreement between birth weight information and medical record data (intraclass correlation coefficient, 0.93 [95% CI, 0.88 to 0.96]).

### Statistical Analysis

All associations between birth weight and NDCs were examined using conditional regression analyses within the generalized estimating equation framework implemented in the drgee package of R (R Project for Statistical Computing). These analyses did not make any distributional assumptions (eg, normal distribution), were appropriate for continuous and binary outcomes, and included calculations of doubly robust SEs.^24,25^ First, we examined the association between the independent variable, birth weight (used as a continuous variable, presented in kilograms), and the dependent variables, categorical neurodivergence (diagnosis of autism, ADHD, or ID) and dimensional neurodivergence (autistic traits, ADHD traits, and IQ), across individuals, while also adjusting for age and sex. In this model, included twins were treated as singletons, not adjusting for familial confounders. Second, we examined within-pair associations between birth weight and categorical or dimensional assessments of NDCs. In this co-twin design, the difference in birth weight within a pair was correlated with the difference in NDCs within the same pair, and we adjusted for all factors shared between twins (genes and shared environment). Third, we repeated the within-pair analyses among the dizygotic and monozygotic twin pairs, respectively. For the dimensional outcome (ie, traits), we used the identity link within the drgee function in R and reported unstandardized regression estimates. For binary outcomes (ie, diagnosis of NDC), we used odds ratios (ORs) with the logit link and present the log ORs in back-transformed format. Information on the generalized estimating equation framework and the twin statistics is presented in the eMethods and the eFigure in Supplement 1. Two-tailed tests with *P* < .05 were considered significant. Data analysis was conducted in November 2022.

## Results

### Participants

This study included 393 participants, with a median age of 15 (range, 8-37) years. There were 230 monozygotic same-sex twins and 159 dizygotic twins (1 triplet); zygosity was unknown for 4 participants. There were 185 female participants (47.1%) and 208 male participants (52.9%). A total of 110 participants (28.0%) had been diagnosed with ADHD, 92 (23.4%) with autism, 20 (5.1%) with ID, 66 (16.8%) with other NDCs (eg, communication disorders, specific learning disorders, or motor disorders), and 102 (26.0%) with other psychiatric disorders (including 46 with depressive disorders and 22 with anxiety disorders). Numbers of pairs concordant and discordant for NDCs, stratified by zygosity, are presented in Table 1. Note that concordant pairs may be discordant at the trait level with within-pair differences in dimensional score points. In the total sample, 78 (19.8%) had a birth weight of less than 2.00 kg and 178 (45.3%) had a birth weight of less than 2.50 kg. The mean (SD) birth weight was 2.49 (0.64) kg for monozygotic twins and 2.55 (0.70) kg for dizygotic twins. Eighteen (monozygotic) twins (4.6%) had a reported TTTS.

**Table 1.** Twin Sample Characteristics

Characteristic	Total twin sample (N = 393)	Sex		Zygosity	
		Female (n = 185)	Male (n = 208)	Dizygotic (n = 159)	Monozygotic (n = 230)
Sex, No. of participants (%)					
Female	185 (47.1)	NA	NA	68 (42.8)	114 (49.6)
Male	208 (52.9)	NA	NA	91 (57.2)	116 (50.4)
Age, median (IQR), y	15 (12-20)	17 (13-25)	14 (11-18)	14 (11-18)	17 (13-24)
Diagnosis, No. of participants (%)					
ADHD	110 (28.0)	43 (23.2)	67 (32.2)	62 (39.0)	46 (20.0)
Autism	92 (23.4)	36 (19.5)	56 (26.9)	41 (25.8)	50 (21.7)
ID	20 (5.1)	8 (4.3)	12 (5.8)	5 (3.1)	15 (6.5)
Other psychiatric disorders	102 (26.0)	59 (31.9)	43 (20.7)	51 (32.3)	48 (20.9)
Qualitative discordance, No. of twin pairs					
ADHD	56	22	34	39	16
Autism	54	21	33	30	24
ID	12	6	6	5	7

Abbreviations: ADHD, attention-deficit/hyperactivity disorder; ID, intellectual disability; NA, not applicable.

### Associations Between Birth Weight and Dimensional (Trait) Neurodivergence Across and Within Twin Pairs

Table 2 shows the models for dimensional (trait-level) neurodivergent outcomes with birth weight as the exposure. Higher birth weight was associated with fewer autistic traits, but not ADHD traits or IQ, across individuals. Further, older participants had lower ratings on autistic traits and ADHD traits and higher IQ ratings. Within the pairs, the association between higher birth weight and fewer autistic traits remained, although only among the monozygotic pairs. In addition, within the monozygotic pairs, higher birth weight was associated with fewer ADHD traits and higher IQ ratings. After twins with TTTS were removed from the analysis, the associations remained for the monozygotic subset for autism traits (unstandardized β [B], −15.77 [95% CI, −27.66 to −3.88]; *P* = .009), ADHD traits (B, −0.24 [95% CI, −0.39 to −0.08]; *P* = .003), and IQ (B, 7.24 [95% CI, 0.41 to 14.07]; *P* = .04).

**Table 2.** Associations Between Birth Weight (in Kilograms) and Trait Neurodivergence

Trait neurodivergence	B (95% CI)			
	Across pairs	Within pairs	Within dizygotic pairs	Within monozygotic pairs
Autism (n = 390)^a^	−5.51 (−10.09 to −0.94)^b^	−13.02 (−23.91 to −2.13)^b^	−9.65 (−26.50 to 7.21)	−17.35 (−28.66 to −6.04)^b^
Sex	−1.62 (−9.17 to 5.92)	NA	NA	NA
Age	−1.63 (−2.15 to −1.12)^b^	NA	NA	NA
ADHD (n = 386)^c^	−0.05 (−0.12 to 0.02)	−0.14 (−0.34 to 0.06)	−0.05 (−0.37 to 0.28)	−0.25 (−0.39 to −0.11)^b^
Sex	−0.04 (−0.13 to 0.07)	NA	NA	NA
Age	−0.03 (−0.04 to −0.02)^b^	NA	NA	NA
IQ (n = 386)^d^	1.12 (−2.27 to 4.51)	3.75 (−2.41 to 9.91)	0.70 (−7.78 to 9.18)	7.43 (1.05 to 13.82)^b^
Sex	−2.65 (−6.79 to 1.50)	NA	NA	NA
Age	0.48 (0.18 to 0.77)^b^	NA	NA	NA

Abbreviations: ADHD, attention-deficit/hyperactivity disorder; B, unstandardized β; NA, not applicable.

^a^ Measured with the Social Responsiveness Scale–2.

^b^ *P* < .05. Male sex was used as the reference.

^c^ Measured with the Child Behavior Checklist or the Adult Behavior Checklist.

^d^ Measured with the Wechsler Intelligence Scale for Children–IV or the Wechsler Adult Intelligence Scale for Adults–IV.

### Associations Between Birth Weight and Diagnostic NDCs Across and Within Twin Pairs

Table 3 shows the models for categorical (diagnosis) neurodevelopmental outcomes with birth weight as an exposure. Higher birth weight was associated with a lower OR for autism and ID, but not ADHD diagnosis, across individuals. Within pairs, the association between higher birth weight and lower odds of autism diagnosis remained; an association with lower odds of ADHD was also observed, although only among the monozygotic pairs. After twins with TTTS were removed from the analysis, the associations between higher birth weight and lower odds of autism still remained (OR, 0.03 [95% CI, 0.001 to 0.54]; *P* = .02), although the association with ADHD diagnosis was lost (OR, 0.007 [95% CI, 0 to 1.10; *P* = .05).

**Table 3.** Associations Between Birth Weight (in Kilograms) and Diagnostic Neurodivergence

Diagnostic neurodivergence	Odds ratio (95% CI) (N = 393)			
	Across pairs^a^	Within all pairs^a^	Within dizygotic pairs^b^	Within monozygotic pairs^c^
Autism	0.63 (0.45 to 0.88)^d^	0.16 (0.03 to 0.81)^d^	0.40 (0.10 to 1.62)	0.02 (0.001 to 0.42)^d^
Sex	0.76 (0.44 to 1.30)	NA	NA	NA
Age	0.93 (0.89 to 0.98)^d^	NA	NA	NA
ADHD	1.16 (0.82 to 1.62)	0.50 (0.12 to 2.01)	0.90 (0.25 to 3.30)	0.003 (0 to 0.70)^d^
Sex	0.84 (0.50 to 1.40)	NA	NA	NA
Age	0.90 (0.85 to 0.96)^d^	NA	NA	NA
ID	0.42 (0.19 to 0.92)^d^	0.05 (0 to 4.28)	Not calculated^e^	Not calculated^e^
Sex	0.76 (0.25 to 2.37)	NA	NA	NA
Age	0.96 (0.89 to 1.04)	NA	NA	NA

Abbreviations: ADHD, attention-deficit/hyperactivity disorder; ID, intellectual disability; NA, not applicable.

^a^ There were 92 participants with autism, 110 with ADHD, and 20 with ID.

^b^ There were 41 participants with autism, 62 with ADHD, and 5 with ID.

^c^ There were 50 participants with autism, 46 with ADHD, and 15 with ID.

^d^ *P* < .05.

^e^ There were too few participants with ID to calculate; male sex was used as the reference.

## Discussion

In this study of phenotyped monozygotic and dizygotic twins enriched for NDCs, we observed that lower birth weight was significantly associated with NDCs. A lower birth weight was foremost associated with more autistic traits as well as an autism clinical diagnosis across individuals. Within the monozygotic pairs, but not the dizygotic pairs, the twin with a higher birth weight had fewer symptoms of autism and ADHD, higher IQ ratings, and lower odds of having a diagnosis of autism and ADHD. These results emphasize birth weight as an NSE factor for NDCs, since the association was most evident when implicitly adjusting for all unmeasured familial confounding (ie, the factors shared between members of the same cluster [twin pair]).

Previous research has reported low birth weight being associated with a wide range of neurodivergent outcomes such as NDCs, including symptoms of autism,^5^ ADHD,^6^ and low IQ.^7^ Partly in line with these findings on singletons, our results across individuals suggested that lower birth weight was associated with autism and ID, but not with a diagnosis of ADHD or traits of ADHD. This finding regarding ADHD contradicts other studies in which low birth weight was associated with ADHD.^6,26^ Yet as indicated in the review by Sciberras et al,^6^ the causal effect of factors such as birth weight on ADHD cannot be confirmed using observational designs compared with twin designs.

The discordant co-twin design offers opportunities to investigate possible causal pathways to NDCs by focusing on the differences within twin pairs with respect to unique environmental experiences, thereby automatically controlling for both genetic factors and shared environment.^27^ In our within-pair analyses, the associations observed were driven by the monozygotic pairs, indicating an NSE factor and genetic contribution to the associations. More specifically, the twin with a lower birth weight had more trait-level and categorical autism and ADHD as well as lower IQ ratings. None of these associations was present among the dizygotic twins. If there were an equal intrapair correlation in monozygotic and dizygotic pairs, the results would suggest NSE factors and no genetic contribution to the association.^9^ Here, the association was only present among monozygotic twins—a reason why the genetic contribution could mask NSE factors in the dizygotic pairs—resulting in a greater association in monozygotic pairs when adjusting for genetic confounding. Previous studies exploring differences in the association between birth weight and dimensional and diagnostic NDCs stratified by zygosity have been few and were mostly conducted with population-based nonclinical phenotyped samples, with mixed findings.^10,12,13,14^ Our findings foremost stress the importance of birth weight as a contributing factor to dimensional and categorical autism and ADHD as well as IQ, but they also acknowledge the importance of genetics for the association between birth weight and NDCs.

Monozygotic twins, compared with dizygotic twins, have a higher risk for adverse birth outcomes, including low birth weight and shorter gestations.^28^ Monozygotic twins often share a single placenta and are connected with each other through vascular anastomoses. Birth weight discordance within monozygotic twins may reflect uneven placental sharing, as in selective fetal growth restriction, and a more unbalanced intertwin blood transfusion (eg, in TTTS) or slow intertwin blood transfusion (eg, twin anemia polycythemia sequence).^21^ Although TTTS may result in a more extreme form of birth weight discordance, our results remained unaltered when pairs with reported TTTS were excluded, indicating that TTTS did not explain the result and that low birth weight is an important factor for NDCs per se.

According to the developmental origins of health and disease (DOHaD) hypothesis, intrauterine processes alter fetal growth program tissue differentiation in a manner that predisposes to different outcomes, including NDCs.^8^ However, as stated by O’Donnell and Meaney,^8^ previous studies failed to account for underlying genetic factors and for the transition of DOHaD from observational studies to those of biological mechanisms, and an integration of genetic information into developmental models is required. In this study, we expanded previous research by providing support for the DOHaD hypothesis within a co-twin control design, adjusting for unmeasured familial confounders. The applied discordant twin design is a powerful method for investigating the contribution of NSE factors to NDCs. Other strengths of this study include its relatively large and rare sample of well-phenotyped twins, concordant and discordant for NDCs.

### Limitations

This study has some limitations. All data about birth weight were based on retrospective parental questionnaires, which may be affected by recall bias. However, a subsample of the data has been validated against medical records data, with excellent agreement.^9^ Other complications during pregnancy, such as preeclampsia, anemia, diabetes, infection, and defective implantation of the placental vessels, may affect birth weight; these were not controlled for, as they are likely to affect both twins comparably, thus not introducing bias. Twin fetuses have a substantially higher risk of low birth weight than nontwins, and a twin pregnancy is also associated with more complications than singleton pregnancies; thus, concerns have been raised that findings in twin samples may not be generalizable to nontwin samples. However, previous research suggests that twins do not systematically differ from the general population of nontwins on measures of behavior and development.^29,30^ Comorbidity was common in our sample, which may have affected the reported associations but also increased the generalizability of our findings, as comorbidity is common in NDCs.^3^ Our sample was biased toward monozygotic twins discordant for NDCs. Although this made it inappropriate to use bivariate twin models such as ACE (A [additive genetics], C [common or shared environment], and E [unique environment]), it was appropriate for investigating the association between NSE factors and outcomes while controlling for familial confounders. Although a co-twin design implies a high degree of genetic and shared environment control, there may be post-twining de novo alterations that may contribute to discordance.

## Conclusions

The findings of this case-control study suggest that as an indicator for fetal growth alterations, lower birth weight is an NSE factor for neurodivergent outcomes, including both trait and categorical autism, ADHD, and ID. Since this association was only observed among the monozygotic twin pairs, a genetic confounding of the association, besides the nonshared factor, was also evident. Given this link between low birth weight and future NDCs, it is important to acknowledge birth weight as influential when assessing NDCs. Furthermore, it is of pivotal importance to facilitate early identification of factors contributing to fetal growth restriction, such as uneven placental sharing, unbalanced intertwin blood transfusion, or slow intertwin blood transfusion, and offer monitoring and treatments (eg, laser treatment) to minimize detrimental outcomes. Future studies are needed to obtain deeper knowledge about the biological mechanisms that underly the associations between low birth weight and NDCs.

## Notes

Supplement 1. **eMethods.** Information About the Statistical Models **eFigure.** Within-Pair Association Between Autistic Traits and Birth Weight in 2 Example Pairs
eReferences

Supplement 2.
Data Sharing Statement
