# Supplementary material for: Evaluation of Birth Weight and Neurodevelopmental Conditions Among Monozygotic and Dizygotic Twins
Source: JAMA Netw Open. 2023 Jun 30;6(6):e2321165. doi: 10.1001/jamanetworkopen.2023.21165 (PMC10314302; doi:10.1001/jamanetworkopen.2023.21165)
Supplement: Supplement 1. — eMethods. Information About the Statistical Models eFigure. Within-Pair Association Between Autistic Traits and Birth Weight in 2 Example Pairs eReferences [file jamanetwopen-e2321165-s001.pdf]

Supplemental Online Content

Isaksson J, Ruchkin V, Ljungström T, Bölte S. Evaluation of birth weight and neurodevelopmental conditions among monozygotic and dizygotic twins. *JAMA Netw Open*. 2023;6(6):e2321165. doi:10.1001/jamanetworkopen.2023.21165

eMethods. Information About the Statistical Models
eFigure. Within-Pair Association Between Autistic Traits and Birth Weight in 2 Example Pairs
eReferences

This supplemental material has been provided by the authors to give readers additional information about their work.

© 2023 Isaksson J et al. *JAMA Network Open*.

eMethods. Information About the Statistical Models

Regression models were fitted using the conditional generalized estimating equations (GEE) framework that accounts for the use of related individuals in the analysis, including calculation of robust standard errors. The model is not limited to distributional assumption (e.g., normal distribution or such) of outcomes and residuals, and the GEE is a recommended analytic approach in co-twin control designs and appropriate for continuous and binary outcomes.[1]

In the across-pair analyses, with linear regression models for estimates of associations between birthweight and NDCs (between-pairs estimates), twins were treated as individuals/singletons, although clustered standard errors were used accounting for the twin correlation. In these analyses, adjustment was made for sex and age.

In the within-pair analyses, conditional linear regression model was used for estimates of association within-pairs (the difference in the exposure variable within a pair is correlated to the difference in the outcome variable within the same pair, see Figure below) after adjusting for factors shared within twins. The within-pair analyses implicitly control for everything shared by the twins within a pair (e.g., shared environment, including family socioeconomic status and parenting styles, as well as on average 50% of segregating genes in DZ-pairs and 100% of genes in MZ-pairs), which is kept constant in the model and do not have an effect on the outcome. There is no need to adjust for gender and age.

The within-pair analyses were further split by zygosity to allow us to further decipher the complexity of genotype-environment-phenotype associations since this allow comparison

© 2023 Isaksson J et al. *JAMA Network Open*.

between the degree of genetics controlled for. The within-pair associations among MZ twin-pairs offers unique opportunities to investigate possible pathways to NDCs by focusing on the within-twin pair differences with respect to unique environmental experiences, i.e., nonshared factors such as birthweight.

© 2023 Isaksson J et al. *JAMA Network Open*.

eFigure. Within-Pair Association Between Autistic Traits and Birth Weight in 2 Example Pairs (Pair 1 Coloured Red and Pair 2 Blue)

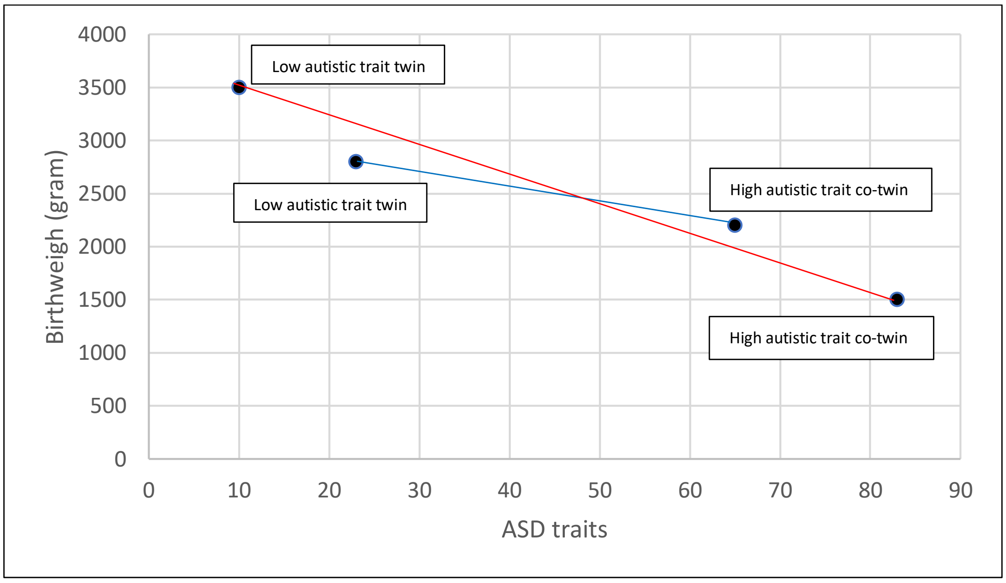

For the continuous outcome (i.e., traits), identity link within the drgee function in R was used, and for binary outcomes (i.e., diagnosis of NDC) the logit link within the drgee function in R was used, and odds ratios are presented in back transformed format.

The conditional GEE is described in Goetgeluk & Vansteelandt article.[2]

© 2023 Isaksson J et al. *JAMA Network Open*.

eReferences

1. Scurrah KJ, Hopper JL. Twin research: Designs and analytic approaches. Conversations in Twins Research. Twins Research Australia. 2019. https://www.twins.org.au/research/tools-and-resources/125-conversation-in-twin-research/377-twin-research-designs-and-analytic-approaches
2. Goetgeluk S, Vansteelandt S. Conditional generalized estimating equations for the analysis of clustered and longitudinal data. Biometrics 2008;64(3):772-80.

© 2023 Isaksson J et al. *JAMA Network Open*.
