# Supplementary material for: Evaluation of Birth Weight and Neurodevelopmental Conditions Among Monozygotic and Dizygotic Twins
Source: JAMA Netw Open. 2023 Jun 30;6(6):e2321165. doi: 10.1001/jamanetworkopen.2023.21165 (PMC10314302; doi:10.1001/jamanetworkopen.2023.21165)
Supplement: Supplement 2. — Data Sharing Statement [file jamanetwopen-e2321165-s002.pdf]

Data Sharing Statement

Isaksson. Evaluation of Birth Weight and Neurodevelopmental Conditions Among Monozygotic and Dizygotic Twins. *JAMA Netw Open*. Published June 30, 2023. doi:10.1001/jamanetworkopen.2023.21165

Data
Data available: No

Additional Information
Explanation for why data not available: The datasets presented in this article are not readily available because of regulations in the ethical approval, requiring among others a data sharing agreement. Requests to access the datasets should be directed to sven.bolte@ki.se
